# Supplementary material for: Establishment of a green fluorescent protein (GFP)-based reporter for picornaviral 3C proteases
Source: J Virol. 2026 Mar 25;100(4):e01936-25. doi: 10.1128/jvi.01936-25 (PMC13098272; doi:10.1128/jvi.01936-25)
Supplement: Supplemental figures — Figures S1 to S6. [file jvi.01936-25-s0001.pdf]

Supporting Information for

Establishment of a green fluorescent protein (GFP)-based reporter for picornaviral 3C proteases

Junki Hirano[1,2], Tsuyoshi Hayashi[3], Yuichi Someya[3], Kazuma Okada[1,2], Kentaro Uemura[1,2], Ming Te Yeh[2,4], Chikako Ono[1,2], Shuhei Taguwa[1,2,4], and Yoshiharu Matsuura[1,2,4]*

Laboratory of Virus Control, [1]Center for Infectious Disease Education and Research (CiDER), [2]Research Institute for Microbial Diseases (RIMD), University of Osaka, Osaka, Japan, [3]Department of Virology II, National Institute of Infectious Diseases, Japan Institute for Health Security, Tokyo, Japan, and [4]Center for Advanced Modalities and DDS (CAMaD), University of Osaka, Osaka, Japan

*Yoshiharu Matsuura
Emails: matsuura@biken.osaka-u.ac.jp

This PDF file includes:

Figures S1 to S6

Supplementary figure 1

A

Name	Sequence
flipGFP (5AA)	TVQ/GP
flipGFP (10AA)	RTATVQ/GPSL
flipGFP (20AA)	KPVLRTATVQ/GPSLDFALSL
flipGFP (30AA)	KQILKKPVLRTATVQ/GPSLDFALSLLRRNI
flipGFP (40AA)	YSGAPKQILKKPVLRTATVQ/GPSLDFALSLLRRNIRQVQT

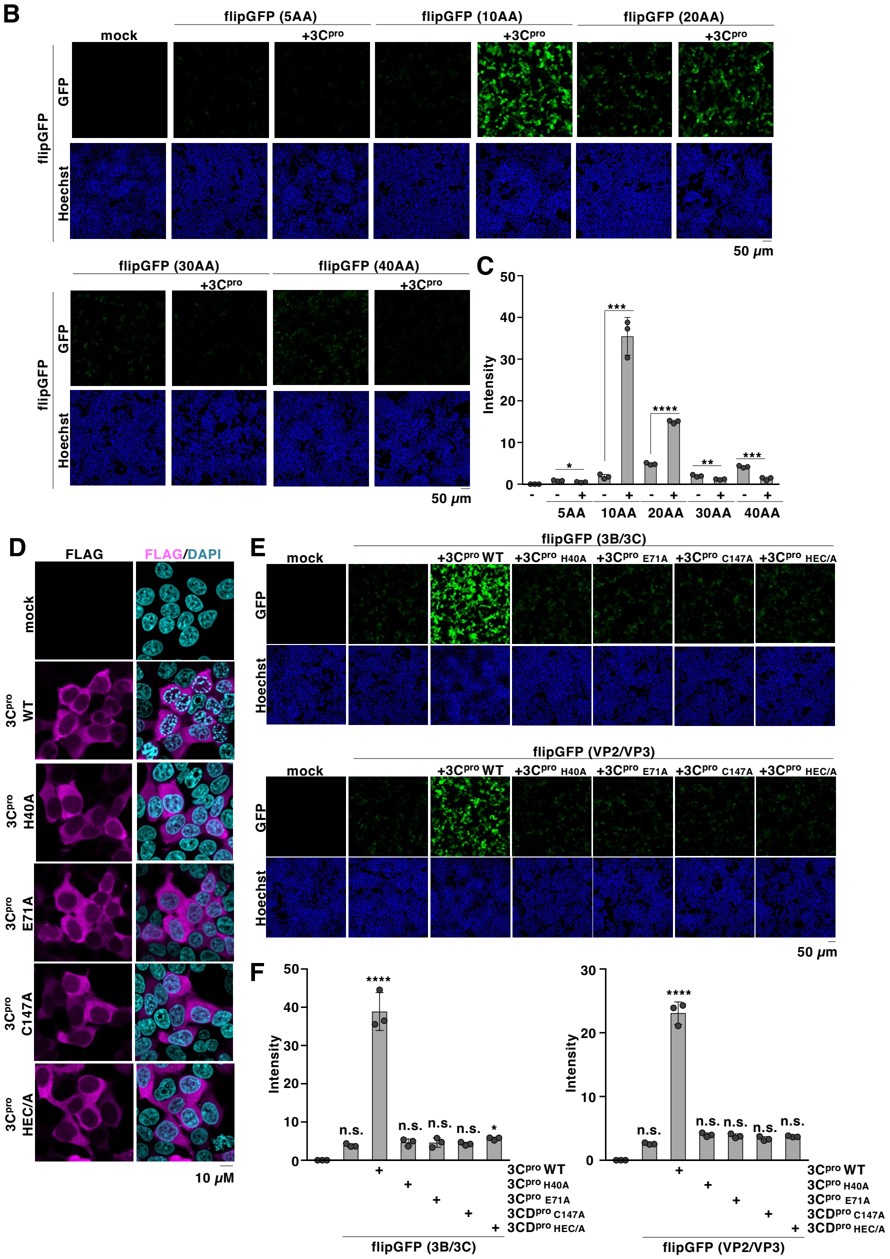

Supplementary figure 1. (A) The 5, 10, 20, 30 and 40 amino acid (AA) sequence between the 3B and 3C region of EV-A71 polyprotein that was inserted into flipGFP. (B) HEK293T cells were transfected with the plasmid encoding 3C protease of EV-A71 and the flipGFP inserted 5, 10, 20, 30 or 40 AA cleavage

sequence. The fluorescent signal of flipGFP was monitored with fluorescent microscopy. (C) HEK293T cells were transfected with the plasmid encoding 3C protease of EV-A71 and the flipGFP inserted 5, 10, 20, 30 or 40 AA cleavage sequence. The fluorescent intensity was calculated 24 h post-transfection. (D) Wild-type or mutant 3C protease was expressed in HEK293T cells. The expression of each 3C protease was detected with fluorescent microscopy using a FLAG antibody. (E) Wild-type or mutant 3C protease was transfected along with flipGFP (3B/3C) or flipGFP (VP2/VP3). The fluorescent signals were detected using microscopy at 24 h post-transfection. (F) Wild-type or mutant 3C protease was transfected along with flipGFP (3B/3C) or flipGFP (VP2/VP3). The fluorescent intensity was calculated 24 h post-transfection. The data presented in B-F are representative of two independent experiments. For the experiment presented in C, significance was determined using Student's t-test (n = 3). For or the experiments presented in F, significance was determined using a one-way ANOVA test (n = 3) (*P ≤ 0.05; **P ≤ 0.01; ***P ≤ 0.001; ****P ≤ 0.0001; n.s., not significant).

Supplementary figure 2

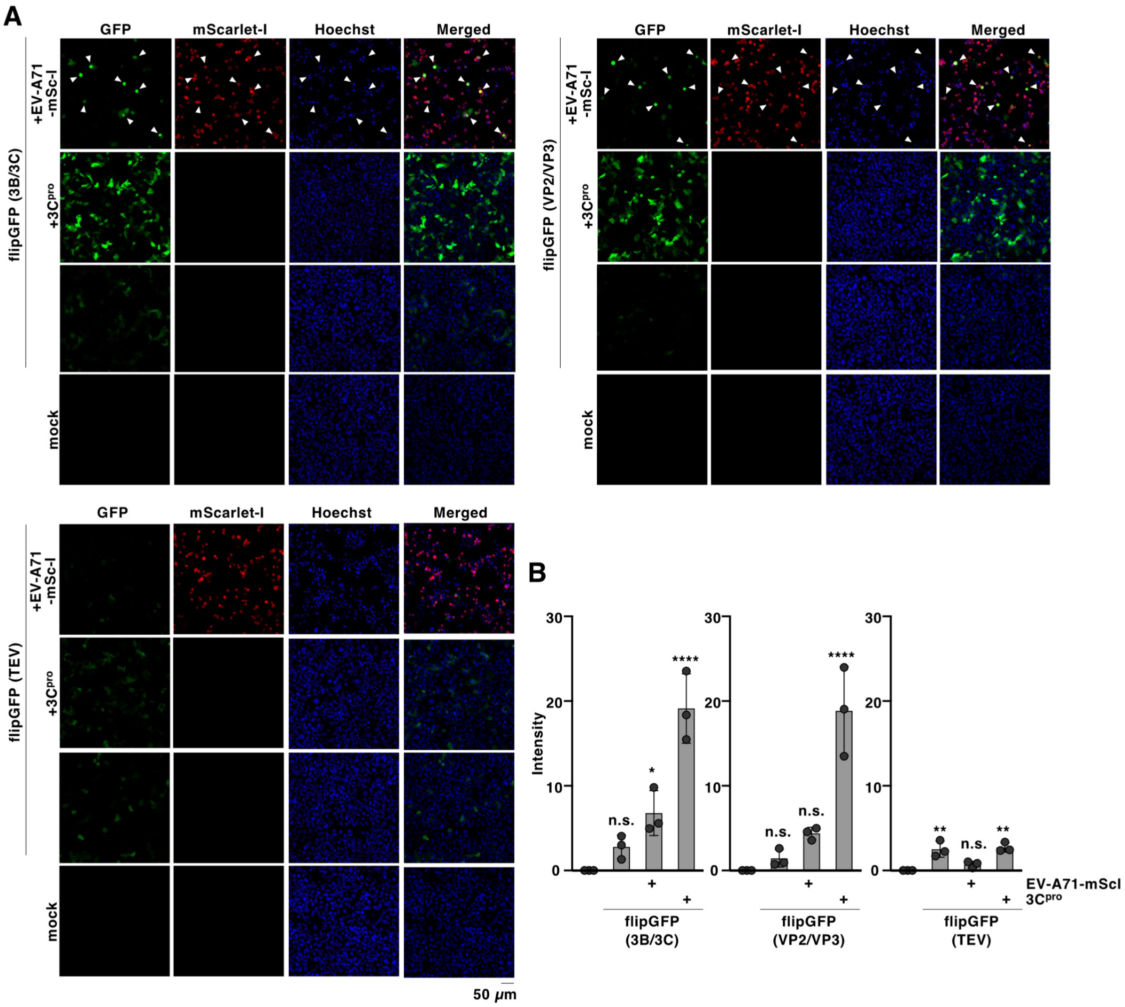

Supplementary figure 2. (A) RD-S cells were transfected with the plasmid encoding flipGFP (3B/3C), flipGFP (VP2/VP3) or flipGFP (TEV) and incubated for 6 h. As a control, 3C protease was transfected along with each flipGFP. The RD-S cells only transfected with flipGFPs were subsequently infected with EV-A71-mScI at an MOI of 1.0. The fluorescent signals were detected using microscopy at 8 h post-

infection. Arrowhead: flipGFP-positive infected cells. (B) RD-S cells were transfected with the plasmid encoding flipGFP (3B/3C), flipGFP (VP2/VP3) or flipGFP (TEV) and incubated for 6 h. As a control, 3C protease was transfected along with each flipGFP. The RD-S cells only transfected with flipGFPs were subsequently infected with EV-A71-mScI at an MOI of 1.0. The fluorescent intensity was calculated 8 h post-infection. The data presented in A and B are representative of two independent experiments. For the experiments presented in B, significance was determined using a one-way ANOVA test (n = 3) (*P ≤ 0.05; **P ≤ 0.01; ****P ≤ 0.0001; n.s., not significant).

Supplementary figure 3

A

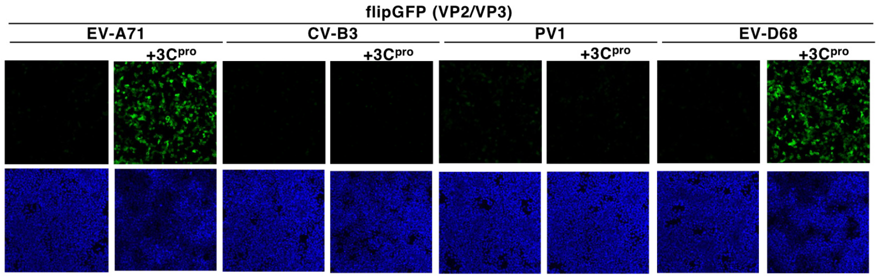

B

Virus	Accession number	Sequence	Virus	Accession number	Sequence
EV-A71	AB550334.1	RQAVTQ/GFPT	PV1	V01150.1	TLPRLQ/GLPV
CV-B3	JX312064.1	RLAGHQ/GLPT	EV-D68	NC_038308.1	RHAITQ/GVPT

C

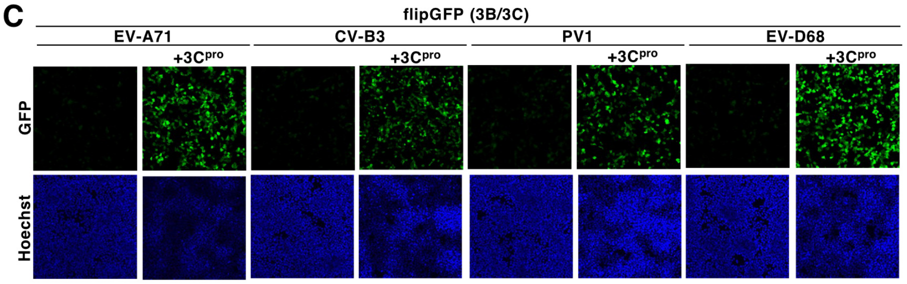

Supplementary figure 3. (A) HEK293T cells were transfected with the plasmid encoding 3C protease derived from genus *Enterovirus* (EV-A71, CVB3, PV1, and EV-D68) and the flipGFP (VP2/VP3). The fluorescent signal of flipGFP was monitored with fluorescent microscopy. (B) The accession number of the *Enterovirus* used in this study and the corresponding amino acid sequence between the VP2 and VP3 regions. (C) HEK293T cells were transfected with the plasmid encoding 3C protease derived from the genus *Enterovirus* (EV-A71, CVB3, PV1, and EV-D68) and the flipGFP (3B/3C). The fluorescent signal of flipGFP was monitored with fluorescent microscopy. The data presented in A and C are representative of two independent experiments.

Supplementary figure 4

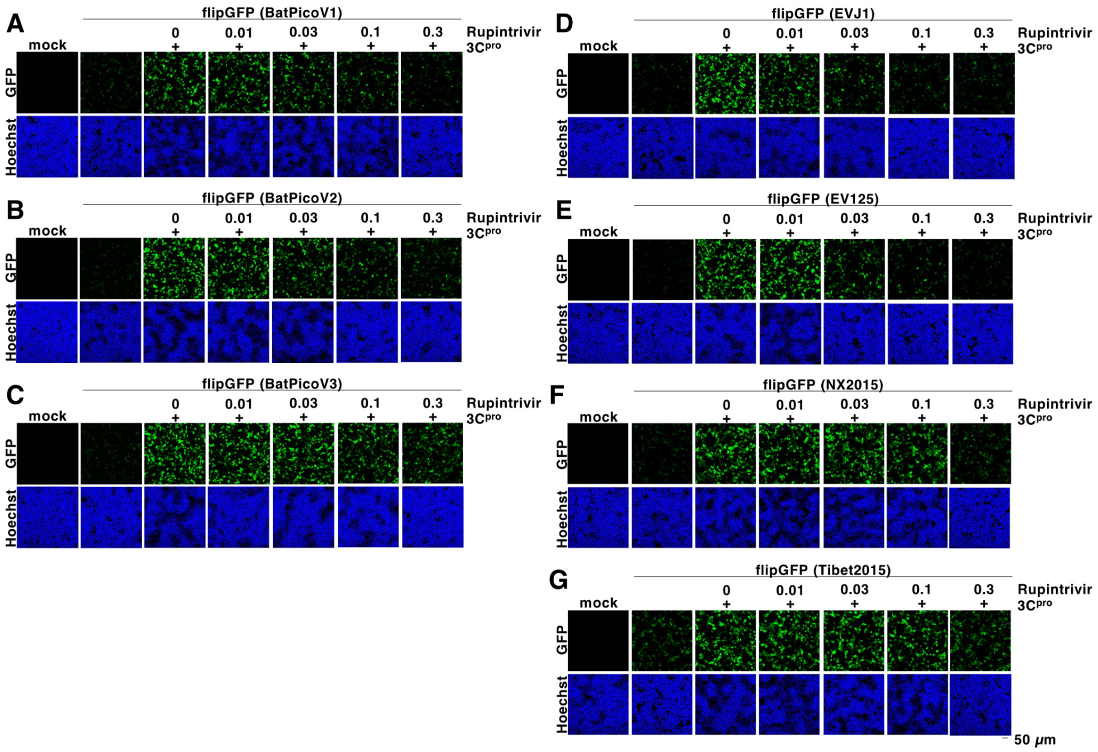

Supplementary figure 4. (A-G) HEK293T cells transfected with the expression vector of 3C protease and flipGFP (BatPicoV1 for Fig. S4A, BatPicoV2 for S4B, BatPicoV3 for Fig. S4C, EVJ1 for Fig. S4D, EV125 for Fig. S4E, NX2015 for Fig. S4F, and Tibet2015 for Fig. S4G) were treated with rupintrivir at concentrations of 0.01, 0.03, 0.1, or 0.3 μM. The fluorescent signals were detected using microscopy at 24 h post-transfection. The data presented in A-G are representative of two independent experiments.

Supplementary figure 5

A

Strain	Nucleotides between the 3B and $3C^{pro}$	$3B/3C^{pro}$
#1	AGAACAGCCACGGTCCAAGGGCCCAGCTTA	RTATVQ/GPSL
#2	CGCACGGCAACAGTACAGGGCCCAAGTCTT	RTATVQ/GPSL
#3	AGAACAGCCACCGTTCAGGGGCCTAGCTTG	RTATVQ/GPSL
#4	CGCACAGCAACAGTGCAGGGCCCGAGCCTT	RTATVQ/GPSL
#5	CGCACGGCAACAGTACAGGGTCCAAGTCTC	RTATVQ/GPSL
#6	AGAACAGCTACTGTTCAGGGACCCAGCCTG	RTATVQ/GPSL
#7	CGTACGGCAACAGTGCAGGGCCCAAGTCTT	RTATVQ/GPSL
#8	CGTACGGCAACTGTGCAGGGGCCGAGCTTG	RTATVQ/GPSL
#9	CGCACGGCAACTGTGCAGGGGCCGAGTTTG	RTATVQ/GPSL
#10	CGCACGGCAACTGTACAGGGGCCGAGCTTG	RTATVQ/GPSL

Strain	Nucleotides between the $3C^{pro}$ and 3D	$3C^{pro}/3D$
#1	TATTTTGCCAGCGAGCAAGGAGAGATCCAG	YFASEQ/GEIQ
#2	TACTTTGCAAGTACACAGGGTGAGATCCAA	YFASTQ/GEIQ
#3	TACTTTGCAAGTGAGCAGGGTGAAATTCAG	YFASEQ/GEIQ
#4	TACTTTGCTAGTGAACAAGGAGAGATCCAG	YFASEQ/GEIQ
#5	TACTTCGCCAGCGAGCAAGGAGAGATCCAG	YFASEQ/GEIQ
#6	TATTTCGTTAGCGAGCAAGGTGAGATCCAG	YFVSEQ/GEIQ
#7	TACTTCGCAAGTGAACAAGGTGAGATCCAA	YFASEQ/GEIQ
#8	TACTTTTGCAGTGAACAAGGTGAGATCCAA	YFCSEQ/GEIQ
#9	TACTTTTGCAGTGAGCAAGGTGAGATCCAA	YFCSEQ/GEIQ
#10	TACTTTTGCAGTGAACAAGGTGAGATCCAA	YFCSEQ/GEIQ

B

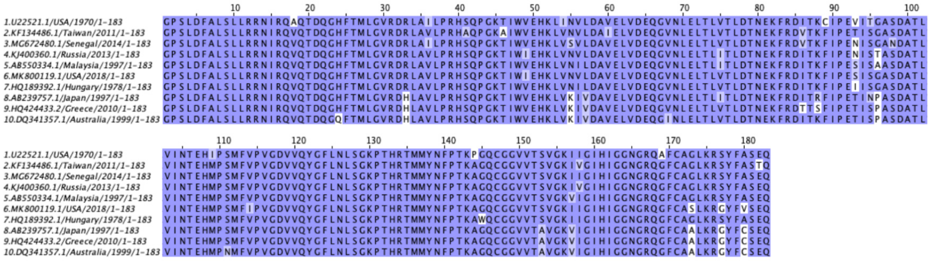

C

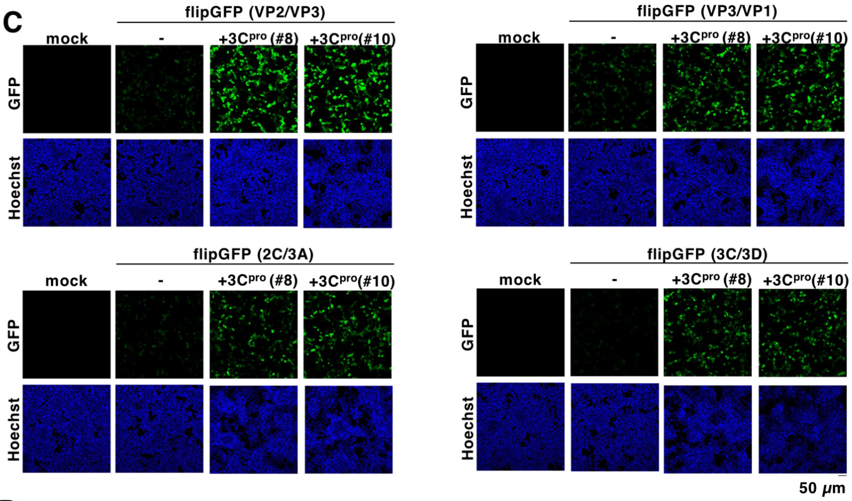

D

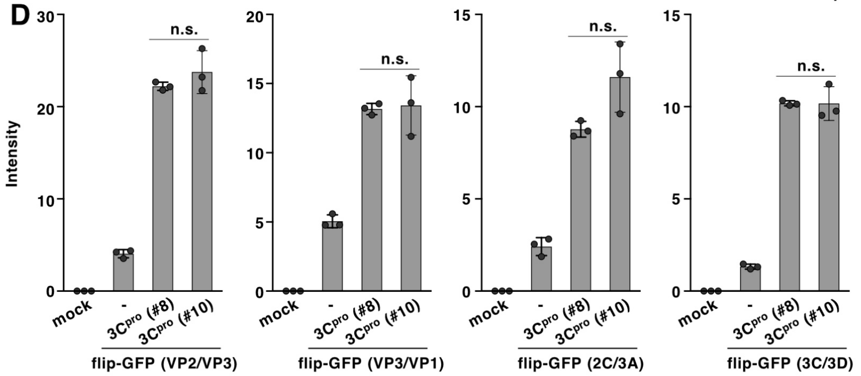

Supplementary figure 5. (A) The examined nucleotide and amino acid sequence between the 3B and 3C regions of EV-A71 (*Left*). The examined nucleotide and amino acid sequence between the 3C protease and 3D regions of EV-A71 (*Right*). (B) The amino acid sequence of EV-A71 3C protease used in this study was aligned by Clustal Omega (EMBL-EBI) and indicated using Jalview software version 2.11.4.1. (C) HEK293T cells were transfected with the plasmid encoding the 3C protease of strain #8 or #10 and the flipGFP inserted sequence derived from the VP2/VP3, VP3/VP1, 2C/3A, or 3C/3D sequence of EV-A71. The fluorescent signals were detected using microscopy at 24 h post-transfection. (D) HEK293T cells were transfected with the plasmid encoding the 3C protease of strain #8 or #10 and the flipGFP inserted sequence derived from the VP2/VP3, VP3/VP1, 2C/3A, or 3C/3D sequence of EV-A71. The fluorescent intensity was calculated 24 h post-transfection. The data presented in D is representative of two independent experiments. For the experiment presented in E, significance (n.s., not significant) was determined using Student's *t*-test (n = 3).

Supplementary figure 6

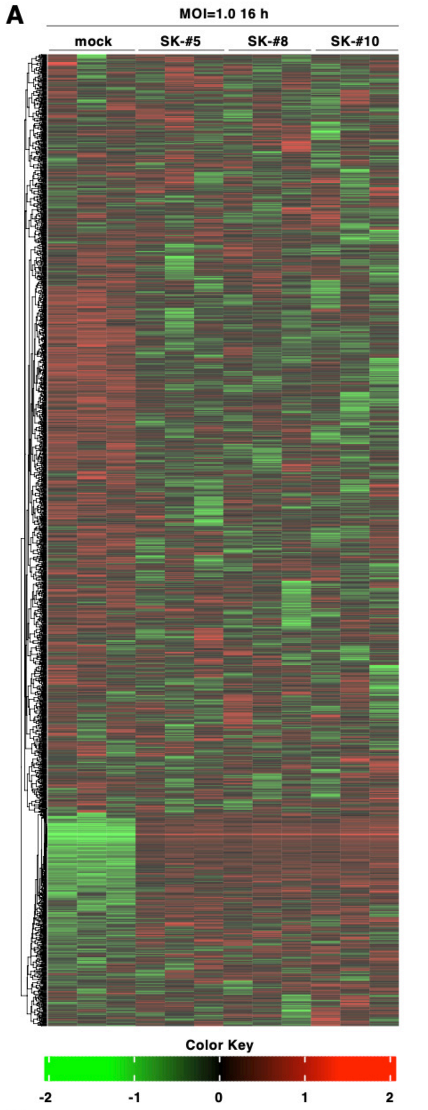

B

Comparison	Pathway	Adj.Pval
SK#5 vs SK#8	No significant pathway found.	n.a.
SK#5 vs SK#10	No significant pathway found.	n.a.
SK#8 vs SK#10	Structural constituent of chromatin	3.4e-02

Supplementary figure 6. (A) RD-S cells were infected with EV-A71 carrying 3C protease of wild type (SK-#5), strain #8 (SK-#8) or strain #10(SK-#10) at an MOI of 1.0, extracted total RNA at 16 h post-infection and analyzed using RNA sequencing (RNAseq). The result of RNAseq was visualized using a heatmap. (B) Gene ontology (GO) molecular function pathways of RD-S cells infected with EV-A71 were compared using Integrated Differential Expression and Pathway (iDEP) analysis. Adj.Pval, adjusted p-value.
